# Dynamic switching of lateral inhibition spatial patterns

**DOI:** 10.1098/rsif.2022.0339

**Published:** 2022-08-24

**Authors:** Joshua Hawley, Cerys Manning, Veronica Biga, Paul Glendinning, Nancy Papalopulu

**Affiliations:** ^1^ Faculty of Biology Medicine and Health, The University of Manchester, Manchester, UK; ^2^ Department of Mathematics, The University of Manchester, Manchester, UK

**Keywords:** lateral inhibition, dynamic switching, spatial pattern, Notch signalling, HES5, neural tube

## Abstract

Hes genes are transcriptional repressors activated by Notch. In the developing mouse neural tissue, HES5 expression oscillates in neural progenitors (Manning *et al.* 2019 *Nat. Commun.* **10**, 1–19 (doi:10.1038/s41467-019-10734-8)) and is spatially organized in small clusters of cells with synchronized expression (microclusters). Furthermore, these microclusters are arranged with a spatial periodicity of three–four cells in the dorso-ventral axis and show regular switching between HES5 high/low expression on a longer time scale and larger amplitude than individual temporal oscillators (Biga *et al.* 2021 *Mol. Syst. Biol.* **17**, e9902 (doi:10.15252/msb.20209902)). However, our initial computational modelling of coupled HES5 could not explain these features of the experimental data. In this study, we provide theoretical results that address these issues with biologically pertinent additions. Here, we report that extending Notch signalling to non-neighbouring progenitor cells is sufficient to generate spatial periodicity of the correct size. In addition, introducing a regular perturbation of Notch signalling by the emerging differentiating cells induces a temporal switching in the spatial pattern, which is longer than an individual cell’s periodicity. Thus, with these two new mechanisms, a computational model delivers outputs that closely resemble the complex tissue-level HES5 dynamics. Finally, we predict that such dynamic patterning spreads out differentiation events in space, complementing our previous findings whereby the local synchronization controls the rate of differentiation.

## 1. Introduction

**Figure 1.**
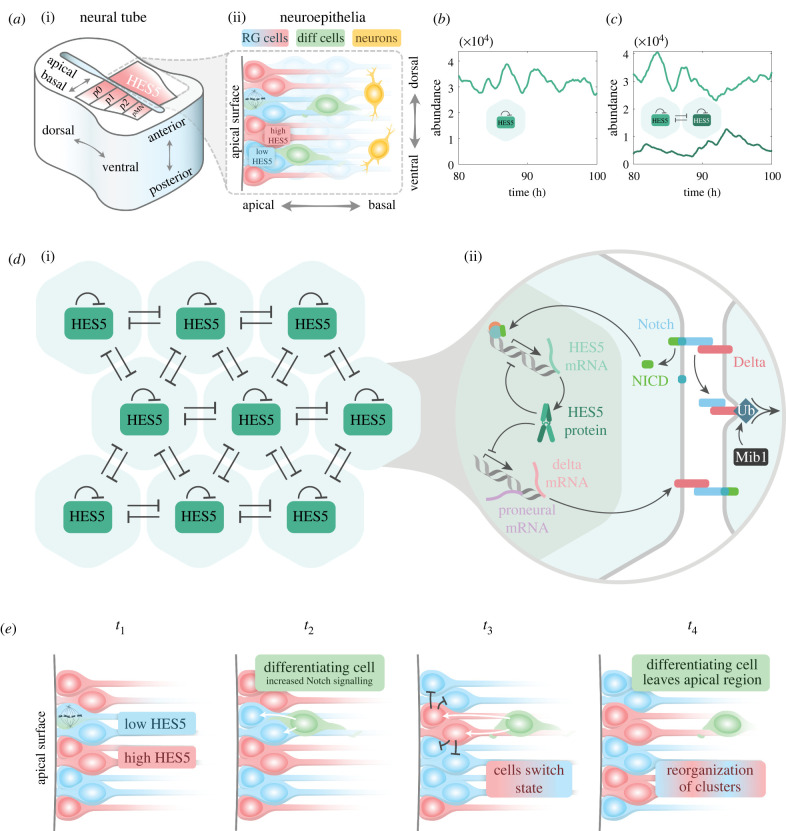
(*a*) (i) Diagram of a neural tube cross section with domains p0–p2 and pMN highlighted, which correspond to where HES5 is expressed. (ii) Structure of the neuroepithelium that makes up the neural tube, with various cell types highlighted. (*b*) Single-cell simulation time trace showing an example of HES5 auto-inhibition producing noisy ultradian oscillations (single-cell parameters in table 1 were used to generate the time trace). (*c*) Time traces for a two-cell simulation with LI coupling (parameters in table 1 used, *τ*_*LI*_ = 0 and *P*_0,*LI*_ = 4500). (*d*) (i) Hexagonal lattice summarizing the interactions in the model, which include nearest-neighbour lateral inhibition and HES5 auto-inhibition. (ii) Detailed interactions of the Notch-HES pathway, which the modelling is based on. Abbreviations: Ub, ubiquitination; Mib1, mindbomb1. (*e*) The proposed mechanism by which differentiating cells could cause a reorganization of the LI spatial pattern by increasing Notch activation in neighbouring cells. At time point *t*_1_, a spatial period is already present as a result of Notch signalling, and an individual cell is shown undergoing mitosis that will give rise to a differentiating cell in *t*_2_. At *t*_2_, a cell with low HES5 commits to differentiation and starts increasing both Delta and Mib1 expression. At *t*_3_, the increased signalling from the differentiating cell causes an increase in the amount of HES5 in the receiving cell, and the differentiating cell starts migrating basally. At *t*_4_, the differentiating cell eventually loses signalling contact with the RG cells at the apical surface, and a reorganized spatial pattern remains.

The developing neural tube is a densely packed pseudostratified neuroepithelium, and starting from E10 in mouse, apically located progenitors called radial glial (RG) cells asymmetrically divide, detach from the apical wall and migrate basally to generate differentiating neuronal cells (figure 1*a*) [1,2]. In specific dorsal–ventral regions of the neural tube, RG cells express the transcriptional repressor HES5 (figure 1*a*,*b*), which maintains cells in a progenitor state by repressing proneural gene expression [3–8]. HES5 expression is dependent on active Notch signalling, which is a pathway that enables contacting cells to signal to each other. Notch signalling can either act to laterally induce expression between cells or laterally inhibit, and this is dependent on the ligand that is interacting with the Notch receptor. If Notch interacting cells express Jagged (1 or 2), then active Notch signalling in one cell will induce active Notch in neighbouring cells. On the other hand, if cells express Delta (1 or 4), then active Notch signalling in one cell will lead to inhibition of active Notch signalling in neighbouring cells. There are further considerations to bear in mind such as *cis*-inhibition where Delta on the same cell binds to and blocks Notch from becoming activated, as is the case with the ligand Delta-3 or high levels of Delta-1/4 [9,10]. In the ventral HES5 domain, the main Notch ligand expressed is Delta-1 [11], and so from here onwards, Notch signalling will refer to Notch/Delta-1 lateral inhibition (LI) (figure 1*c*).

**Table 1.** Model parameter values used [14].

symbol	value	biological definition
*a*_*m*_	0.77 min^−1^	transcription rate
*a*_*p*_	26 min^−1^	translation rate
*u*_*m*_	ln(2)/30 min^−1^	mRNA degradation rate
*u*_*p*_	ln(2)/90 min^−1^	protein degradation rate
*P*_0,auto_	25 000 proteins	HES5 auto-inhibition repression threshold
*P*_0,*LI*_	0–10 000 proteins	LI coupling repression threshold
*n*_auto_	3.5	HES5 auto-inhibition Hill coefficient
*n*_*LI*_	3	lateral inhibition Hill coefficient
*τ*_auto_	30 min	HES5 self-repression time delay
*τ*_*LI*_	0–100 min	lateral inhibition time delay

This study investigates HES5 expressed in the p0–p2 and pMN domains of the neural tube, which are distinct progenitor domains that give rise to different neuronal subtypes (figure 1*a*) [12]. It was previously known that individual cells are capable of oscillatory HES5 dynamics due to transcriptional auto-inhibition [13] with a temporal period of around 3.3 h [3], which we will refer to as ultradian oscillations. Around 50% of cells in the HES5 domain are found to exhibit oscillatory behaviour and otherwise have aperiodic noisy expression [3]. Two recently uncovered key aspects of HES5 expression observed in *ex vivo* slices of neural tube are first, that within the p0–p2 domains, HES5 is expressed in clusters of similar expression (groups of 3–7 cells), and these are arranged regularly to form an average spatial period of three to four cells measured along the dorsal–ventral axis [14]. Second, the location of high and low expression is not fixed over time, with clusters of similarly expressing cells spending an average of around 8 h in a high or low state before switching to the opposite state. The ultradian oscillations have a mean temporal period of 3.3 h as well as a smaller amplitude than the observed 8 h switching behaviour, indicating that ultradian oscillations alone are not responsible for the longer switching behaviour. Indeed, both noisy and oscillatory single-cell HES5 dynamics are found to be nested within the larger amplitude switching behaviour of the cells when looking at individual cell traces [14]. Specifically, the amplitude of the longer switching dynamics is approximately twice that of the ultradian amplitude as determined previously [3].

Most theoretical models of Notch LI produce stationary patterns where cells do not switch between high and low states once the spatial pattern has formed [15–21]. Some literature explores anti-phase oscillations of downstream Notch genes between coupled cells, but this concerns ultradian oscillations only, rather than ultradian oscillations nested within a distinct larger amplitude, longer time scale switching behaviour [22,23]. Therefore, the dynamic switching behaviour of the HES5 spatial pattern, as far as we are aware, is not accounted for in the literature. To simulate neural tube HES5 dynamics, Biga *et al.* [14] used a multi-cellular Notch-HES5 model composed of parametrized single-cell dynamics that were coupled together via LI interactions, signalling only between closest neighbours (figure 1*d*). This work considered that ultradian HES5 oscillations may interact via LI to generate an emergent behaviour similar to that observed in the neural tube. Aspects such as local synchronization of HES5 dynamics could be reproduced; however, other aspects of the data such as three- to four-cell spatial periodicity and larger amplitude temporal switching could not be reproduced, indicating that additional mechanisms are required to explain the observed patterns of dynamic behaviour.

To understand the complexity and generation of the *ex vivo* neural tube pattern, we consider two new additions to the multi-cellular Notch-HES5 model presented in the study by Biga *et al*. [14]. The first addition is extending the LI signalling distance between cells, inspired by the modelling work that shows how protrusions can extend Notch signalling distance, which leads to longer period spatial patterns [18]. This is in line with experimental observations of filopodia in *Drosophila*, which have been shown to carry Notch ligands and induce Notch signalling several cell diameters away [17,24], and various literature points to the existence of protrusions in the neuroepithelia that are probably capable of Notch signalling [25–28]. The second addition to the model is the introduction of a differentiation process that alters the amount of Notch signalling that neighbouring cells receive from a differentiating cell. This process in the model is based on the fact that early differentiating cells migrating out of the RG population increase their expression of Delta [29] as well as Mindbomb1 (Mib1), which greatly increases the efficiency of Delta trans-activation of Notch [30–32]. Via ubiquitination, Mib1 marks Delta for endocytosis, which subsequently provides the mechanical force required for successful Notch receptor activation on neighbouring cells [30,33] (figure 1*e*).

To identify outputs similar to *ex vivo* dynamics in the new model, we use significance testing on power spectra to identify spatial periodicity and define a new measure, the dynamicity coefficient, to indicate the proportion of time cells spend in high and low states. By plotting these measured outputs in parameter space, we identified that extended signalling distance generates spatial periods of three to four cells, and the inclusion of a differentiation process that dynamically alters signalling between cells produced switching behaviour between high and low HES5 expression over time. In addition, the model output showed cases of ultradian oscillations nested within the larger amplitude, longer time-scale switching behaviour, as observed in single-cell data [14]. This is a unique exploration of how Notch LI signalling can be prevented from permanently settling into fixed peak and trough locations while maintaining the spatial pattern forming ability of LI. The reorganization of peak and trough locations of HES5 is found to enable differentiation events to be spread out spatially over time and prevents hotspots where differentiating cells are repeatedly produced, potentially important in ensuring an even production of neurons across the dorsal–ventral axis.

## 2. Methods

### 2.1. Multi-cellular lateral inhibition HES5 model

**Figure 2.**
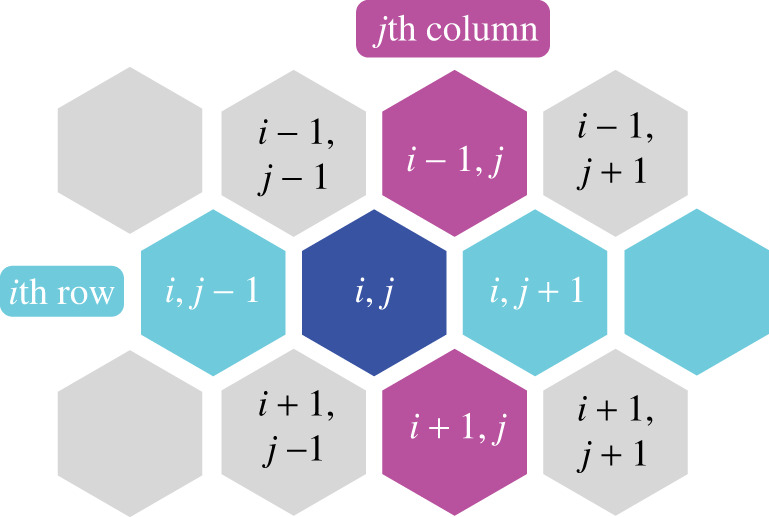
Hexagonal geometry of the model and how the *i* and *j* indices in the model equations map onto this grid.

Our core model is based on previously implemented modelling work, consisting of auto-inhibition interactions of HES5 protein back on to expression of its own mRNA, and with HES5 dynamics being coupled between cells in a hexagonal geometry using an inhibitory Hill function representative of Notch LI [16,34]. The single-cell parameters used in the model were previously parametrized to neural tube HES5 data using Bayesian inference [3], and a range of multi-cellular parameters were explored in [14]. Figure 1*d* outlines the biological interactions considered and the core interactions that are described mathematically in the model. A chemical Langevin equation approach is used [35], and the stochastic delay differential equations that govern the dynamics of a cell at row *i* and column *j* (see figure 2) in the multi-cellular model are given by

2.1 $$\begin{array}{rl} \frac{dm_{ij}(t)}{dt}= & -\mu_{m}m_{ij}(t)+\alpha_{m}H_{\mathrm{auto}}(\;p_{ij}(t-\tau_{\mathrm{auto}}))\\ & \;H_{LI}({\bar{\;p}}_{ij}(t-\tau_{LI}))+\eta_{m} \end{array}$$

and

2.2 $$\frac{dp_{ij}(t)}{dt}=-\mu_{\;p}p_{ij}(t)+\alpha_{\;p}m_{ij}(t)+\eta_{p},$$

where *m*_*ij*_(*t*) is HES5 mRNA concentration in the cell on the *i*th row and *j*th column at time *t* and *p*_*ij*_(*t*) is HES5 protein concentration. *μ*_*m*_ and *μ*_*p*_ are the degradation rates of HES5 mRNA and protein, respectively; *α*_*m*_ and *α*_*p*_ are the transcription and translation rates; *τ*_auto_ is the time delay associated with HES5 autorepression; and *τ*_*LI*_ is the time delay associated with the lateral inhibition interaction between cells. In Results §3.2, we find that over a range of *τ*_*LI*_ values (0–100 min), the model exhibited a similar behaviour. The somitogenesis literature points to a range of possible *τ*_*LI*_ values (20–120 min), and so to reduce the complexity of the model, we set *τ*_*LI*_ = 0 for the main results and give an exploration of non-zero time delays in electronic supplementary material, figures S2 and S3 [36–40].

Both the functions *H*_auto_ and *H*_*LI*_ are inhibitory functions that regulate mRNA production rate in response to protein abundance either within a cell (*H*_auto_), or between cells (*H*_*LI*_). *H*_*LI*_ therefore is the coupling function that enables HES5 dynamics to influence neighbouring HES5 dynamics. For the signalling contribution from each cell in contact with a receiving cell, we take the approach used in [16,23], and the amount of HES5 a cell receives is the averaged abundance from all signalling neighbours

2.3 $${\bar{\;p}}_{ij}=\frac{1}{\left|N(i,j)\right|}\sum_{\left(i,j)\in N(i,j\right)}\varepsilon_{i,j}p_{ij},$$

where N(i,j) is the set of neighbours a cell is in signalling contact with and |N(i,j)| is the total number of neighbours in the set. Neighbouring cells are defined in §3.1, and this introduces proximal and distal cells. Proximal cells are adjacent cells, i.e. any neighbours within a one-cell distance, and distal cells are signalling neighbours that lie further than one-cell distance away (see figure 6). Coupling strength of distal and proximal cells, *ɛ*_*d*_ and *ɛ*_*p*_ respectively, can be varied independently in the model, and so *ɛ*_*i*,*j*_ in equation (2.3) defines a coupling weighting depending on whether the signalling neighbour is proximal or distal and can have one of two values

2.4 $$\varepsilon_{i,j}=\left\{ \begin{array}{cc} 1,\; & \text{if}\;N(i,j)\;\text{ is a proximal neighbour}\;\\ \frac{\varepsilon_{d}}{\varepsilon_{p}},\; & \text{if}\;N(i,j)\;\text{is a distal neighbour.}\; \end{array}\right.$$

The Hill functions are both decreasing functions, where *H*_auto_(0) = *H*_*LI*_(0) = 1 and *H*_auto_(∞) = *H*_*LI*_(∞) = 0 and have the form

2.5 $$H_{\mathrm{auto}}(\;p_{ij}(t-\tau_{\mathrm{auto}}))=\frac{1}{1+{(\;p_{ij}(t-\tau_{\mathrm{auto}})/P_{0,\mathrm{auto}})}^{n_{\mathrm{auto}}}}$$

and

2.6 $$H_{LI}({\bar{\;p}}_{ij}(t-\tau_{LI}))=\frac{1}{1+{({\bar{\;p}}_{ij}(t-\tau_{LI})/P_{0,LI})}^{n_{LI}}}.$$

*P*_0,auto_ and *P*_0,*LI*_ are the repression thresholds of each Hill function. The repression threshold defines the amount of protein that results in a 50% reduction in mRNA production rate. For example, within an individual cell, the value of *P*_0,auto_ defines the abundance of HES5 protein, *p*_*ij*_, at which mRNA production rate will be 50% within that same cell. In the case of *P*_0,*LI*_, this defines when mRNA production in a receiving cell will be 50% in response to the averaged incoming abundance of HES5 protein in the neighbouring cells ${\bar{\;p}}_{ij}$. *n*_auto_ and *n*_*LI*_ are the Hill coefficients that define how steep the gradient of the Hill function is at *P*_0_ (higher values give a sharper transition between no repression and repression).

The terms *η*_*m*_ and *η*_*p*_ in equations (2.1) and (2.2) are the stochastic noise terms for mRNA and protein, which are Gaussian white noise scaled by the square root of the number of events that occur in each process

2.7 $$\eta_{m}=\sqrt{\mu_{m}m_{ij}(t)+\alpha_{m}H_{\mathrm{auto}}(\;p_{ij}(t-\tau_{\mathrm{auto}}))H_{LI}({\bar{\;p}}_{ij}(t-\tau_{LI}))}\xi_{m}(t)$$

and

2.8 $$\eta_{p}=\sqrt{\mu_{\;p}p_{ij}(t)+\alpha_{\;p}m_{ij}(t)}\xi_{p}(t),$$

where *ξ*_*m*_(*t*) and *ξ*_*p*_(*t*) are Gaussian white noise with mean of 0 and variance of 1, respectively. Equations are solved using the Euler–Maruyama method, implemented in Matlab. Model parameters are summarized in table 1.

### 2.2. Extracting spatial signals from the model

**Figure 3.**
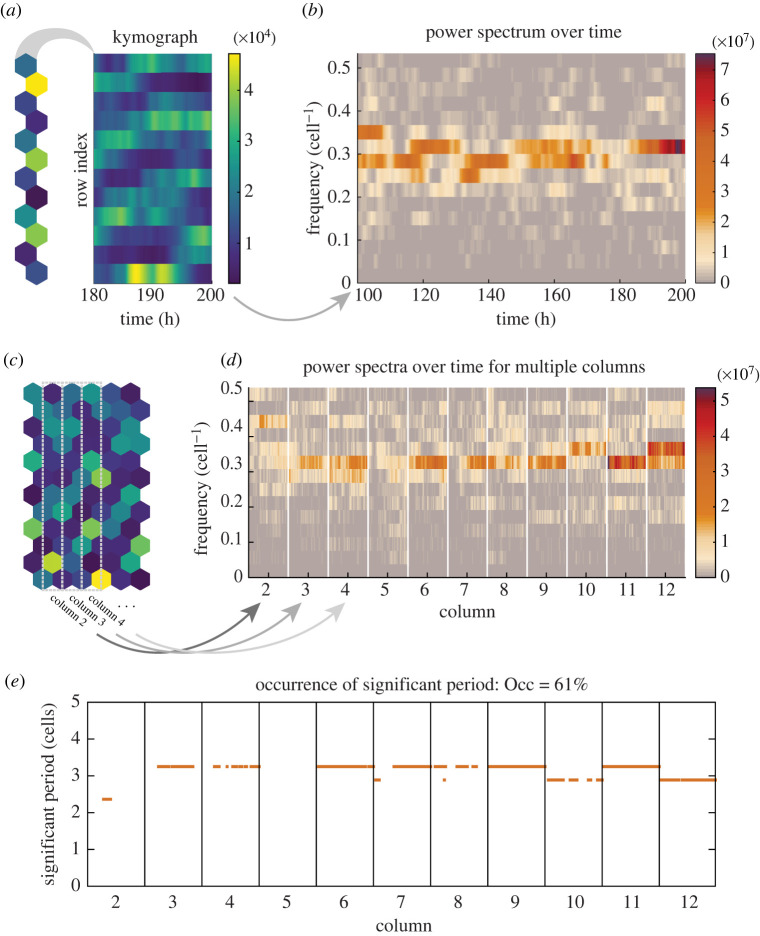
Outline of the process used for detecting the presence of significant periodic spatial patterns. (*a*) In the case of one-dimensional simulations, kymographs are generated from the expression on each row (*y*-axis) and over time (*x*-axis). (*b*) A Fourier transform of the spatial signal in *a* is shown as a power spectrum over time, where colour indicates the power contribution of each frequency at each time point. (*c*) For two-dimensional simulations, spatial signals are generated from the cells that fall within selection regions (grey dashed boxes). (*d*) A power spectrum is generated for the spatial signal in each column (within each division the power spectrum over time is given, like in *b*). (*e*) The peaks of each individual power spectra are tested for significance using the Fisher *g*-test (§2.3) and the significant spatial periods are plotted with orange dots and the occurrence measure is shown above the plot (Occ is defined in §2.3).

To understand what sort of spatial patterns are being produced by the model, we extract spatial signals using a similar approach to that used for the *ex vivo* analysis in [14]. In the hexagonal grid of cells, *p*(*x*_*i*,*j*_, *t*_*k*_) = *p*_*ij*_(*t*_*k*_) denotes that the protein expression at the *i*th row, *j*th column and *k*th time-step, and *I*, *J*, *K* are the total number of rows, columns and time-steps. In the case of simulating a single column of cells (*J* = 1) such as in figure 3*a*, a spatial signal can be generated for each time point by taking the protein expression along the entire column such that the spatial signal at time-step *k* and *j* = 1 is expressed as follows:

2.9 $$S_{k}(x)=p(x_{1\;:\;I,1},t_{k}).$$

Each spatial signal is therefore a vector of length *I*, where each entry is the expression from an individual cell, and the total number of spatial signals that can be generated from a single simulation is *N*_*s*_ = *K* (figure 3*b*). To visualize both spatial and temporal aspects of the data in one plot, the spatial signal can be plotted over a range of time points $S_{k_{1}:\;k_{2}}(x)$ as a kymograph, shown in figure 3*a*,*c*.

In the case of simulating multiple columns (i.e. a two-dimensional grid of cells), we extract the spatial signal by using a selection region with a width of one cell and length *I* cells (see grey dashed boxes in figure 3*c*). Due to the hexagonal geometry, on the even rows, two cells fall within this selection region, and so the spatial signal is constructed as follows:

2.10 $$S_{\;jk}(x_{i})=\left\{ \begin{array}{cc} p(x_{i,j},t_{k}),\; & \text{if}\;i\;\text{odd}\;\\ \frac{\;p(x_{i,j},t_{k})+p(x_{i,j-1},t_{k})}{2},\; & \text{if}\;i\;\text{even}\; \end{array}\right.,$$

where even rows use the average of the two cells that fall within the selection region. As there are multiple columns, the number of spatial signals generated from a single simulation is *N*_*s*_ = *K*(*J* − 1) (figure 3*d*).

### 2.3. Detecting spatial periodicity

From the extracted spatial signals, a method is needed to detect the presence of statistically significant spatial periodicity in the inherently noisy model outputs. By using a fast Fourier transform method (Matlab), a power spectrum can be obtained for each spatial signal

2.11 $$P(S_{\;jk}(x))=|F(S_{\;jk}(x)){|}^{2},$$

where $F(S_{\;jk}(x))$ is the Fourier transform of the spatial signal. The highest power frequency in the power spectrum indicates the dominant periodicity in the spatial signal (figure 3*b*,*d*). To distinguish if the detected peak is due to noise or genuine periodicity, a Fisher’s *g*-test is implemented that compares the peak value in the power spectrum with the sum of the whole power spectrum and is defined as follows:

2.12 $$g=\frac{P(\omega_{\mathrm{peak}})}{\sum_{n=1}^{N/2}P(\omega_{n})},$$

where $P(\omega_{n})$ is the power/contribution from the *n*th frequency *ω* analysed in the Fourier transform. The *g*-value tends to 1 in the case of genuine periodic signals and 0 for noisy/aperiodic signals. To determine significance, a p-value can be calculated by comparing the likelihood of obtaining a higher *g*-value than the observed *g* if the power spectrum was generated from a purely noisy signal *S*_*ξ*_(*x*). This formally would be

2.13 $$p_{\mathrm{val}}=P(g_{\xi }>g|H_{0}),$$

where *g*_*ξ*_ is the expected *g*-value obtained from *S*_*ξ*_(*x*), and *H*_0_ is the null hypothesis. In this case, the null hypothesis is that the power spectrum is generated by Gaussian white noise, for which an analytical calculation of *P*(*g*_*ξ*_ > *g*| *H*_0_) is given in [41,42]. Spatial signals with *p*_val_ < 0.05 are accepted as having significant periodicity present. In addition, we define occurrence to be the fraction of all the spatial signals analysed (over all columns over all time points) that are found to have significant periodicity

2.14 $$\mathrm{Occ}=\frac{N_{s}^{\;p_{\mathrm{val}}<0.05}}{N_{s}}.$$

Figure 3*e* shows an example of the significant periods detected in a two-dimensional simulation, with the Occ value given.

### 2.4. Distinguishing dynamic and stationary spatial patterns

To develop a mathematical measure of how stationary or dynamic a pattern is over time, the dynamicity coefficient is defined here, which measures the proportion of time the expression in an individual cell spends in a high versus low state.

The choice of threshold that defines high and low states for the dynamicity measure is driven by our desire to analyse the dynamics at all levels of expression. As coupling strength is increased, the mean population expression reduces, but the signal is still dynamic. Therefore, an absolute threshold cannot be used to measure dynamicity across a range of parameters. Instead, we define a relative threshold for each simulation by using the mean population expression level to ensure the threshold lies between the high and low states generated by lateral inhibition. While this ensures a high and low state is always defined, it comes with the caveat that when LI is too weak to produce distinct high and low states, the dynamicity coefficient is reflecting switches due to the noisy fluctuations and ultradian oscillations of HES5.

**Figure 4.**
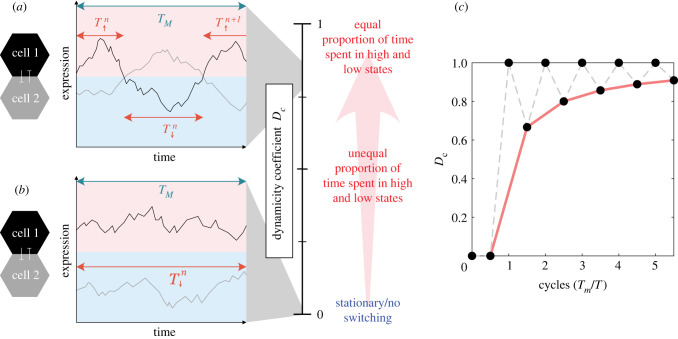
Illustrative examples and plot of the dynamicity coefficient. *T*_*M*_ is defined as the total measurement time/the length of the signal analysed and $T_{↑,↓}^{n}$ denotes the time spent above or below the mean population expression. (*a*) Plot of expression in two individual cells (black and grey) that exhibit dynamic LI, where each cell can switch between a high state (pink upper half of the graph) and low state (lower pale blue half). (*a*) Corresponds to a high *D*_*c*_ value. (*b*) Two cells exhibiting a stationary LI interaction where one cell remains high (black) and one cell remains low (grey) over the whole measurement time. (*b*) corresponds to *D*_*c*_ = 0. (*c*) *D*_*c*_ values that would be obtained for a perfectly switching (regular period) signal at integer and half cycles (black dots). Half cycle measurements are joined with a red line.

The amount of time spent in an individual high or low state, which we call persistence time, is denoted by *T*_↑,*n*_ and *T*_↓,*n*_ respectively, where *n* is the *n*th occurrence of a high or low state (see figure 4*a*). Therefore, the proportion of time a cell spends in a high state in a signal of length *T*_*M*_ (the measurement time) is expressed as follows:

2.15 $$\alpha_{↑}=\frac{1}{T_{M}}\sum_{n=1}^{N}T_{↑,n},$$

where *N* is the total number of occurrences of the cell being in a high state. Similarly, the proportion of the measurement time that a cell spends in a low state is expressed as follows:

2.16 $$\alpha_{↓}=\frac{1}{T_{M}}\sum_{n=1}^{N}T_{↓,n}.$$

As *α*_↑_ and *α*_↓_ are proportions of the total measurement time, their values lie between 0 and 1, and *α*_↑_ = 1 − *α*_↓_. If *α*_↑_ = *α*_↓_ = 0.5, then this implies that an individual cell spends equal amounts of time in high and low states. In the opposite case where either *α*_↑_ = 1 and *α*_↓_ = 0, or *α*_↑_ = 0 and *α*_↓_ = 1, this implies that the cell spends the entire measurement time in one state and therefore is classed as a stationary signal (figure 4*b*). By using these proportions of time spent in high and low states, we define here the dynamicity coefficient as follows:

2.17 $$D_{c}=2\times \min\left(\alpha_{↑},\alpha_{↓}\right),$$

which rescales the proportions to give a value between 0 and 1: 0 if the signal/patterning is stationary and 1 if the signal spends equal amounts of time in the high and low state (*α*_↑_ = *α*_↓_ = 0.5). To prevent transient fluctuations above or below the population mean contributing to the *α*_↑_, *α*_↓_ values, a Savitzky–Golay filter (inbuilt Matlab function) was used to smooth the signal first, using polynomial order of 1 and frame length of 165 minutes [43].

Another property that is useful to extract from the data is how frequently cells switch between high and low states. Here, we define the persistence time as how long the signal persists in a high or low state, which is just the mean time spent high or low

2.18 $${\bar{T}}_{↑}=\frac{1}{N}\sum_{n=1}^{N}T_{↑,n}$$

and

2.19 $${\bar{T}}_{↓}=\frac{1}{N}\sum_{n=1}^{N}T_{↓,n}.$$

In the case of regular temporal switching between states, i.e. an oscillator with a well-defined, non-varying period, then the period of the oscillator is given by $T={\bar{T}}_{↑}+{\bar{T}}_{↓}$.

Note that the measurement time, *T*_*M*_, will affect the *D*_*c*_ value to some extent, depending on how comparable *T*_*M*_ is to the expected period of switching *T*. Take for example a perfectly switching signal where *α*_↑_ = *α*_↓_ = 0.5, if we define the number of measured cycles *n*_*c*_ = *T*_*m*_/*T*, then when *n*_*c*_ is integer, *D*_*c*_ = 1. However, when the measurement time does not coincide with a full cycle, i.e. *n*_*c*_ is non-integer, then *D*_*c*_ < 1, because *α*_↑_ ≠ *α*_↓_. *α*_↑_ and *α*_↓_ will be most different and therefore *D*_*c*_ values will reach a minimum at half cycles, and for a perfectly switching signal, the *D*_*c*_ value at half cycles (*n*_*c*_ = 0.5, 1.5, 2.5, …) is

2.20 $$D_{c}=\frac{2(n_{c}-\frac{1}{2})}{2(n_{c}-\frac{1}{2})+1}.\;$$

This effect of measurement time cutting off at non-integer cycles only becomes a significant issue when *n*_*c*_ is on the order of a few cycles, and so the longer the measurement time relative to the switching period, the more accurate the *D*_*c*_ value becomes (see figure 4*c*).

### 2.5. Implementation of the differentiation-based perturbation algorithm

**Figure 5.**
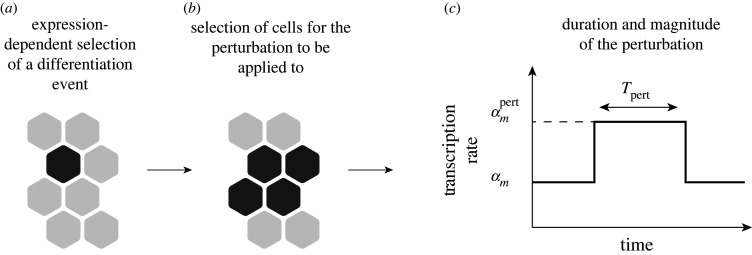
Outline of the differentiation-based perturbation (DBP) algorithm. (*a*) The probability-dependent differentiation algorithm is used to first identify a region where a cell is most likely to differentiate (single dark grey hexagon) based on HES5 expression levels (equation (2.21)). (*b*) An area around the selected cell in *a* is then selected (four dark grey hexagons), and these cells will have the perturbation applied to the HES5 transcription rate with magnitude $F_{\mathrm{pert}}=\alpha_{m}^{\mathrm{pert}}/\alpha_{m}$ for a period *T*_*pert*_ shown in *c*.

To implement a process in the model that represents the signalling strength changes due to cell differentiation, we first require a process to determine the locations of differentiating cells and then a process to apply a changed signalling strength to the area around the differentiating cell. For differentiation, we make use of our previously developed differentiation algorithm [14]. This decision process is based on the assumption that lower levels of HES5 are more likely to enable the upregulation of proneural genes, and therefore, cells are marked for differentiation in a probabilistic manner based on expression (figure 5*a*). The probability of a given cell to be marked for differentiation is given by

2.21 $$P(\mathrm{diff}|p_{ij}(t))=\left\{ \begin{array}{cc} 0,\; & p_{ij}(t)>D_{\mathrm{thresh}}\;\\ R\left(\frac{D_{\mathrm{thresh}}-p_{ij}(t)}{D_{\mathrm{thresh}}}\right),\; & p_{ij}(t)<D_{\mathrm{thresh}},\; \end{array}\right.$$

where *D*_thresh_ is the differentiation threshold (set as the population mean expression), and *R* is the rate of differentiation. For further details on the differentiation algorithm, see [14].

In the neural tube, differentiating cells delaminate from the apical side in a process called apical abscission [2], and migrate outward towards the basal side [44]. However, cell movement is not modelled here, and so differentiation events have to represent something less than the full picture of cell differentiation. Here, we model the effects of a changed signalling strength coming from a differentiating cell in a given area, rather than simulating the differentiating cell explicitly as a separate cell. This involves choosing a number of cells around the area of the already-selected differentiation location that are defined to be in contact with a differentiating cell in that area (figure 5*b*). A perturbation is then applied to the HES5 transcription rate, *α*_*m*_, in these cells to reflect increased Notch activation in these cells (figure 5*c*). The magnitude of the perturbation applied to *α*_*m*_ is determined by a perturbation factor *F*_pert_, so the perturbed HES5 transcription rate is $\alpha_{m}^{\mathrm{pert}}$, where

2.22 $$\alpha_{m}^{\mathrm{pert}}=F_{\mathrm{pert}}\alpha_{m}.$$

Finally, a period of time for the perturbation to be applied is denoted by *T*_pert_ (figure 5*c*). Values for *T*_pert_ and *F*_pert_ are discussed in §3.2.

In the single-column simulation, if cell *i* is the location where a differentiation event is chosen, then the perturbation is applied to both cell *i* and with equal probability either cell *i* − 1 or *i* + 1. If it is a grid simulation, then the perturbation is applied to a group of four cells: always *i*, *j* and *i*, *j* + 1 and then either the two cells above (*i* − 1, *j* and *i* − 1, *j* + 1) or below (*i* + 1, *j* and *i* + 1, *j* + 1). The entire process described in this section is referred to as the differentiation-based perturbation (DBP) algorithm.

Differentiation rates in the simulations are characterized in two different ways. First, the average rate of differentiation over the whole population is calculated as the number of differentiation events that occur per hour as a percentage of the total number of cells in the simulation. Second, to look at how differentiation rates vary in individual cells, the frequency of differentiation per cell is calculated as the number of differentiation events per hour for every cell and then the distribution can be visualized in a histogram (figure 10*d*–*f*).

## 3. Results

### 3.1. Extended signalling distance generates the correct spatial periodicity

**Figure 6.**
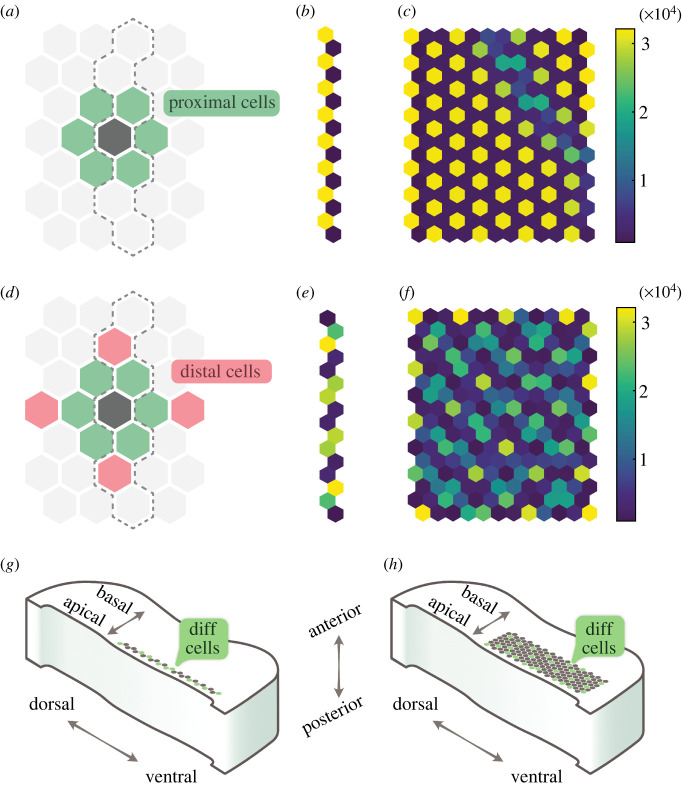
Comparison of deterministic model outputs with and without distal cells (*a–f*), and the mapping of the model lattice to the neural tube (*g*,*h*). (*a*) A cell (dark grey) and the cells it is coupled to in a hexagonal grid in the case when only proximal cells (green) are considered, and the dashed outline indicates the interactions included for one-dimensional simulations. (*b*,*c*) The final time point (200 h simulation) of a one- and two-dimensional simulation, respectively, using the interactions shown in (*a*). (*d*) The extended interactions used for all subsequent simulations, which includes the distal cells (red) in addition to the proximal cells (green). (*e*,*f*) The final time point (200 h simulation) of a one- and two-dimensional simulation, using *ɛ*_*d*_/*ɛ*_*p*_ = 1.5. All simulations shown used *P*_0,*LI*_ = 4000. (*g*) The one-dimensional lattice represents a line of cells in the dorsal–ventral direction towards the apical side of the tissue. Green cells indicate differentiating cells. (*h*) The two-dimensional model lattice maps to the dorsal–ventral, apical–basal plane of the tissue.

Previous work found that the largest spatial period that can be generated from a HES5-Notch model with nearest-neighbour coupling is a two-cell period of alternating high and low expression shown in figure 6*a*–*c* [14]. However, HES5 expression in the neural tube exhibits clusters of similarly expressing cells that are arranged to generate a higher spatial periodicity of three to four cells; therefore, an additional mechanism is required. Given that extending Notch signalling distance has been shown to enable longer spatial periodicity [18,45,46], and protrusions have been observed in many neuroepithelial tissues [17,25–28], we add *distal* cell interactions to represent the longer range protrusion-based signalling (figure 6*d*–*f*).

The distal geometry shown in 6*d* was found to generate sufficiently clustered patterns of the correct spatial period of three to four cells (figure 6*e*,*f*), whereby groups of neighbouring cells form areas of high or low expression. In the one-dimensional case (figure 6*e*), distal cell interactions enable a clear alternating pattern of two cells high and two cells low. In the two-dimensional case (figure 6*f*), the periodic repeating also extends in the second dimension, and a mixture of cluster sizes is shown in figure 6*f*.

**Figure 7.**
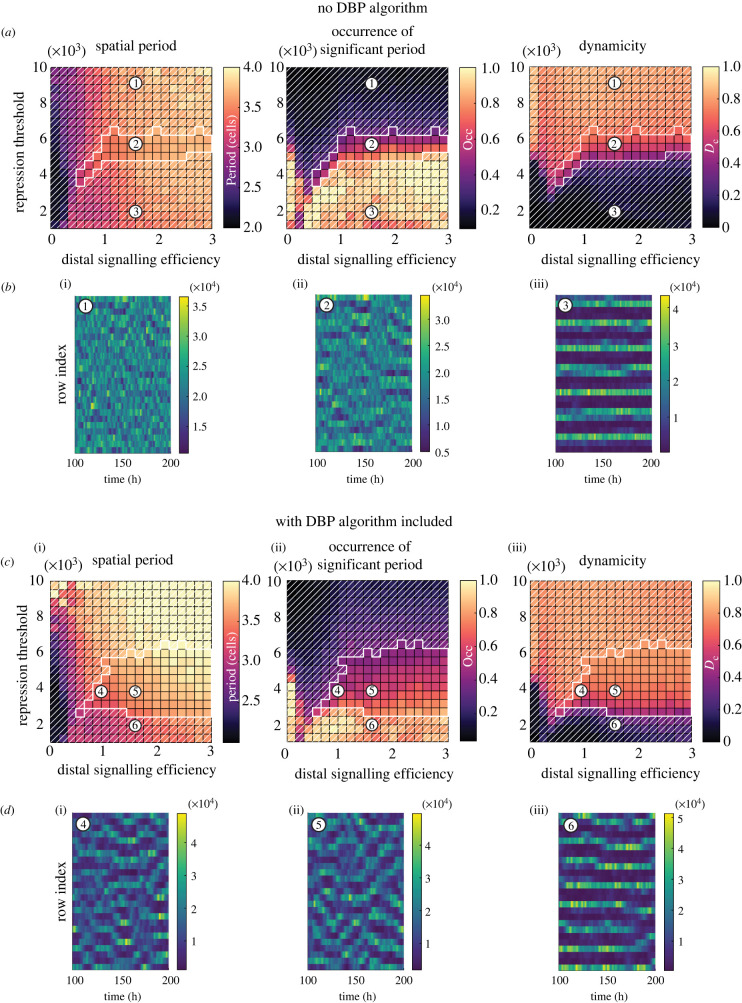
Simulations of a single column of cells (26 rows, 1 column). Parameter space values were generated by taking the mean value from 20 simulations with random initial conditions. The top two rows show model output without the DBP algorithm, and the bottom two rows show the output with DBP included. (*a*) and (*c*) Parameter spaces show distal signalling efficiency, *ɛ*_*d*_/*ɛ*_*p*_, versus lateral inhibition repression threshold, *P*_0,*LI*_, and three different measured model outputs are indicated by the colour scale. Areas without white diagonal lines overlaid indicate regions that satisfy equation (3.1) (*T*_spatial_ > 3, Occ > 0.4, *D*_*c*_ > 0.4), which is where dynamic spatial patterns occur. (i) Spatial period where only statistically significant periods are plotted (see §2.3). (ii) Occurrence of significant spatial period (see §2.3). (iii) The dynamicity measure (see §2.4). (*b*) and (*d*) Example kymograph plots from corresponding numbered regions in parameter space. Kymographs used the following parameters: (1) *P*_0,*LI*_ = 9053, *ɛ*_*d*_/*ɛ*_*p*_ = 1.58, (2) *P*_0,*LI*_ = 5737, *ɛ*_*d*_/*ɛ*_*p*_ = 1.58, (3) *P*_0,*LI*_ = 1947, *ɛ*_*d*_/*ɛ*_*p*_ = 1.58, (4) *P*_0,*LI*_ = 3842, *ɛ*_*d*_/*ɛ*_*p*_ = 0.95, (5) *P*_0,*LI*_ = 3842, *ɛ*_*d*_/*ɛ*_*p*_ = 1.58, and (6) *P*_0,*LI*_ = 1947, *ɛ*_*d*_/*ɛ*_*p*_ = 1.58.

**Figure 8.**
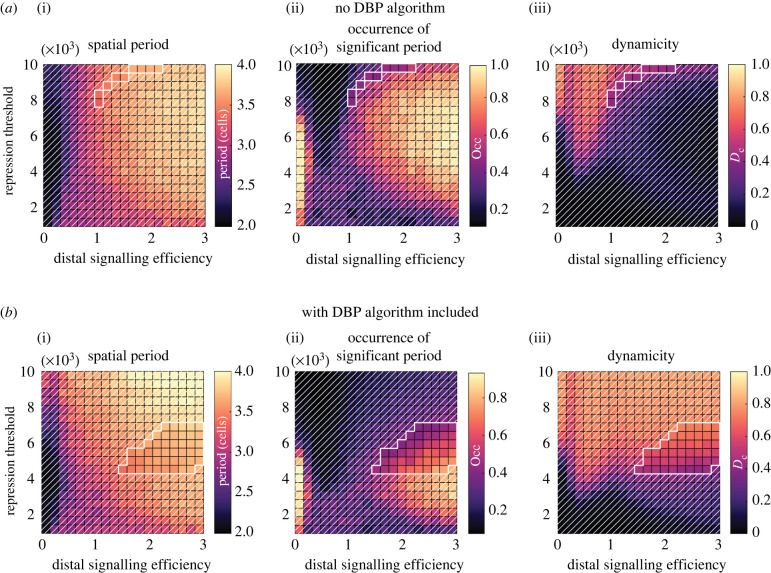
The same parameter spaces as in figure 7 are plotted here but for two-dimensional simulations (26 rows, 6 columns). Parameter space values were generated by taking the mean value from 20 simulations with random initial conditions. (*a*) Model output when the DBP algorithm is not included, and (*b*) is with the DBP algorithm included. Areas without white diagonal lines overlaid indicate regions that satisfy equation (3.1) (spatial period > 3, occurrence of spatial period > 0.4 and a dynamicity coefficient > 0.4), and where dynamic spatial patterns are expected to occur.

HES5 clusters *ex vivo* are composed of three to seven cells on average and are elongated in the apical–basal direction [14]. Although no quantification is done on cluster size in this model, the high expressing clusters can be seen to be more of the order of two to four cells in size in 6*f*. The cluster sizes being lower than *ex vivo* measurement is probably due to there being no elongation of clusters in the model, and it is not known what causes the elongation *ex vivo*. Despite the apical–basal elongation not being reproduced, the dorsal–ventral spatial period of three to four cells is reproduced in the model, which is shown in the spatial period analysis in figures 7 and 8. Other distal geometries were explored and simulations with higher numbers of neighbours produced less clustered or less robust patterns (electronic supplementary material, figure S1). For all subsequent simulations, the distal geometry shown in figure 6*d* is used.

Inspired by modelling work done by [18], proximal and distal signalling strength can be varied relative to each other and distal signalling efficiency here is given by *ɛ*_*d*_/*ɛ*_*p*_, where *ɛ*_*d*_ is the distal coupling strength (LI repression threshold) and *ɛ*_*p*_ is the proximal coupling strength. The effect of distal signalling efficiency is explored in the next section.

The one-dimensional simulations represent a strip of cells in the dorso-ventral direction in the neural tube, towards the apical side where progenitor cells are located and differentiating cells are born (figure 6*g*). The two-dimensional simulations map on to a dorsal–ventral, apical–basal plane (figure 6*h*). In both cases, this is to match the location of the HES5 domain and the axes that have been studied experimentally. In §3.3, differentiating cells are allowed to appear anywhere in the two-dimensional lattice, and so for the dorsal–ventral, anterior–posterior plane assumption to make sense, differentiating cells would need to be distributed across the apical–basal direction of the HES5 domain *ex vivo*. This is probably the case, as differentiating cells are born in the apical domain and subsequently migrate basally (as illustrated in figure 1*e*) and so will probably contact a number of different cells throughout the apical–basal axis. If this is not the case and differentiating cells have a spatial distribution in a particular apical–basal area, then future work could look at the effects of restricting where these differentiating cells appear in the model.

### 3.2. Differentiation expands the dynamic patterning regime in one-dimensional simulations

In addition to the periodic expression of three to four cells, the clusters of similar expression exhibit dynamic switching between high and low expression *ex vivo*. The DBP algorithm described in §2.5 outlined the process through which differentiating cells perturb neighbouring progenitor cells through altered signalling strength. We present the model both with and without the DBP algorithm included to compare which model is more consistent with the dynamic switching observed *ex vivo*. This first set of results uses one-dimensional simulations of 26 rows by 1 column, as this roughly matches the number of nuclei within the dorsal–ventral HES5 expression domain (figure 6*g*) as seen in [14].

To analyse the output of the model, three measurements were plotted in parameter space of distal signalling efficiency (*ɛ*_*d*_/*ɛ*_*p*_), versus the LI repression threshold (the coupling strength between cells) (figure 7). Without DBP (figure 7*a*), the spatial period tends towards two cells at zero/low distal signalling efficiency and towards higher periodicity as distal signalling increases. Occurrence of a significant spatial period (§2.3) becomes more likely at lower repression threshold values (stronger coupling strength). However, as occurrence increases, the *D*_*c*_ value decreases, indicating that the spatial patterns forming are largely stationary ones.

Regions of the parameter space are defined to be a good match to the experimental observations and exhibit dynamic spatial patterns if the spatial period *T*_spatial_, occurrence Occ and dynamicity coefficient *D*_*c*_ satisfy

3.1 $$T_{\mathrm{spatial}}>3,\;\mathrm{Occ}>0.4,\;\mathrm{and}\;D_{c}>0.4.$$

Despite the DBP algorithm not being present, there are perturbations due to expression noise and ultradian oscillations. It is these inherent variations in expression that cause the thin dynamic pattern region of parameter space in figure 7*b* (region without white diagonal lines overlaid), when the coupling strength is sufficiently strong to induce a weak spatial pattern, but weak enough for the inherent perturbations to enable dynamic switching between high and low states. See electronic supplementary material, movie S1 for an animated one-dimensional simulation without DBP included.

For simulations that include the DBP algorithm, we estimated values for *T*_pert_ and *F*_pert_ from the literature. Experimental work in the chick neural tube found that Tis21, a marker for neurogenically dividing neural progenitors, was upregulated around 8 h after Delta-1 was expressed in the same cell, followed by the generation of neurons around 16 h [47]. Assuming that within this time, signalling from the Delta-1 expressing cell upregulates Notch signalling in neighbouring cells, then at least 8h perturbation seems reasonable, and we choose a value of *T*_pert_ = 7 h. In electronic supplementary material, figures S2 and S3, an exploration of other *T*_pert_ values found that longer perturbation enhanced the range of parameters in which dynamic patterning occurred. To estimate a value for *F*_pert_, we searched for literature that explores upregulation of Mib1 and its effect on neighbouring cell’s Notch response, as Mib1 determines the efficiency of transactivation of Notch signalling. One study looked at the effect of co-culturing mouse neocortex Mib1-positive intermediate progenitors and Mib1-negative RG cells with Notch1 expressing cells and compared the resulting levels of Hes1 expression within these Notch1 cells [31]. Two methods were used to isolate populations of Mib1-positive cells, and it was found that compared with RG cells, Mib1-positive cells caused between a 1.8- to 3.7-fold increase in Hes1 expression. In an *in vivo* study of chick neural tube, upregulation of both Delta-1 and membrane-localized Mib1 caused a 1.8-fold increase in HES5 intensity in RG cells [32]. We chose a value from the higher range of these Mib1-induced increases in HES and set *F*_pert_ = 3.

With the DBP algorithm included in the simulation (figure 7*c*,*d*), the parameter space for spatial period and occurrence remains broadly similar. However, high *D*_*c*_ values extend further into lower repression thresholds (high coupling strength) when compared with the simulations without DBP (figure 7*a*,*b*). This generates an expanded region of parameter space that satisfies equation (3.1) (region without white diagonal lines overlaid in figure 7*c*). The kymographs *d*(4) and (5) further confirm and visualize how the dynamic pattern evolves, showing a more definite and higher amplitude spatial pattern forming (compared with *b*(2)) but still with switching of peaks and troughs, and even some transient travelling wave type behaviour. At very low repression threshold *d*(6), stationary patterns still emerge when the perturbation strength cannot overcome the effects of strong coupling. See electronic supplementary material, movie S2 for an animated one-dimensional simulation with DBP included.

We explored the effect of varying the LI time delay between cells. The literature indicates a wide range of possible values this could take, from 20 to 120 min [36–40], and in the electronic supplementary material, figures S2 and S3, *τ*_*LI*_ values between 0 and 100 min are tested, along with *T*_pert_ values between 0 and 14 h. The results show that as *τ*_*LI*_ increases, the dynamic patterning region decreases, but crucially the region still expands when DBP is included, indicating robustness of the proposed mechanism. This reduced dynamic patterning region at longer LI time delays can be expanded by increasing the duration of *T*_pert_, which is the time that a differentiating cell exerts higher signalling on its neighbours. As the expanding effect of DBP inclusion was found to occur across all LI time delays, and due to uncertainty in the actual value of the time delay, we chose to reduce the complexity of the model for the main results and use a value of *τ*_*LI*_ = 0 min.

Taken together this set of results indicates that from the underlying stationary pattern formed by LI, the pattern can be made dynamic by the introduction of perturbations to HES5 levels. The perturbations drive individual cells away from the two attractor states generated by LI (higher/lower phenotype between signalling neighbours) and thus enable opportunities for the reorganization of peak and trough locations. The ability to switch states is a balance between coupling strength and perturbation size; at higher coupling strengths, perturbations cannot change the abundance of HES5 enough to enable a switch between low/high abundance in their neighbours. Conversely, at weak coupling strength, the system has no spatial pattern forming ability. Between these extremes lies a region where lateral inhibition is strong enough to form spatial patterns if unperturbed but capable of switching states given sufficient perturbation.

### 3.3. Dynamic spatial patterning occurs in two-dimensional simulations

To explore how introducing more signalling neighbours affects the dynamic pattern forming ability of the model, we simulate a two-dimensional hexagonal grid and show that the one-dimensional results of the previous section extend to the two-dimensional case. A grid size of 26 rows by 6 columns is chosen as this corresponds approximately to the number of nuclei within the dorsal–ventral, apical–basal HES5 expression domain (figure 6*h*) [14]. The two-dimensional arrangement involves an additional four proximal and two distal neighbours compared with the one-dimensional simulations, meaning that each cell receives an average input signal generated from a larger number of neighbours.

Without DBP included (figure 8*a*), the parameter space outputs were generally similar to the one-dimensional case (figure 7*a*). However, the dynamicity was found to be largely low in the region where high occurrence of a spatial period was found, indicating that only stationary patterns can be generated. The absence of robust dynamic patterning is confirmed by the thin band where equation (3.1) is met (region without a white diagonal line overlay in figure 8*a*), much smaller than in the one-dimensional case. This appears to be due to the additional signalling from the higher number of cells (10 signalling neighbours in the two-dimensional case versus four neighbours in the one-dimensional case), which reinforce the strength of lateral inhibition, making switching states due to stochastic noise and HES5 oscillations less likely. In addition, at low repression threshold values/high coupling strength (0–2000 in figure 8*a*), occurrence was found to be lower than in the one-dimensional case. In this region, LI is found to still drive expression to high and low states as expected; however, due to the higher number of neighbours, the increased signalling strength resulted in irregular patterns, causing a spread of frequency values in the power spectrum, and therefore, no single significant peak detected using the Fisher *g*-test.

When the DBP algorithm is included (figure 8*b*), dynamic patterning is recovered, and a region of high dynamicity is found to overlap with high occurrence of a spatial period. Comparing the acceptance region that satisfies equation (3.1) in figure 8*b* with the one-dimensional case in figure 7*d*, it can be seen that the area is reduced and shifted slightly towards higher distal signalling efficiencies. However, in the one-dimensional case, where distal signalling efficiencies of 1 were found to generate dynamic patterning, the minimum efficiency required in two dimensions is *ɛ*_*d*_/*ɛ*_*p*_ > 1. This need for higher distal signalling is due to the fact that when there are higher numbers of neighbours, individual cells contribute less of an effect on their neighbours due to the incoming signal being the averaged expression of the neighbours. See electronic supplementary material, movie S3 for an animated two-dimensional simulation with DBP included.

As protrusions have a much smaller contact area than when two-cell bodies contact, one might intuitively expect more Notch signalling to occur at the cell body. However, one study identified that not only is contact area an important consideration but that also the diffusion rate of Notch and Delta across the cell membrane influences the amount of signalling that occurs. Higher diffusion rates of Notch and Delta, probably enabled by active transport within protrusions, can in fact enable higher rates of Notch signalling in protrusions than in a larger contact area of cell body with lower diffusion rates of Notch/Delta [48]. In other modelling work, Hadjivasiliou *et al.* suggested the ability to mechanically pull on Delta and subsequently activate Notch signalling may be different at the cell body versus the protrusions [18]. They hypothesized that this difference could be caused by either a reduced amount of endocytosis at the cell body or that the dynamic extending/retracting nature of protrusions could provide the mechanical force required for Notch activation. A final possibility is that *cis*-inhibition may be more common in the cell body than in protrusions, and this would occur if high amounts of both Notch and Delta are present on the cell body, but only one of Notch or Delta is found in the protrusions.

As discussed in the previous section, when DBP is not present in the one-dimensional simulations, the noisy fluctuations and ultradian oscillations are capable of producing a thin region of parameter space where weak dynamic patterning can occur (figure 7*a*). This prompts the question: to what extent do these smaller amplitude fluctuations affect switching when DBP is included? Without DBP, dynamic patterning occurs at weaker LI coupling strengths, where the two high and low attractor states are not sufficiently attracting to prevent noise/ultradian oscillations from inducing switches between the two states. However, at stronger coupling strengths, the LI states attract more strongly, and make smaller amplitude fluctuations increasingly unlikely to induce switching between states when compared with the larger amplitude perturbations provided by DBP (figures 7*c* and 8*b*). This does not rule out that noise/ultradian oscillations play some role in enhancing switching, but it is clear from this analysis these smaller amplitude fluctuations are not sufficient to induce switching at stronger coupling strengths.

### 3.4. Ultradian oscillations are nested within the larger amplitude DBP switching behaviour

*Ex vivo* observations from the study by Biga *et al.* [14] indicate that the temporal dynamics of single cells consists of both noisy and ultradian oscillations (average temporal period of 3.3 h) nested within larger amplitude, longer time scale switching behaviour (average time spent high or low was 8 h). Through measuring persistence times and plotting single-cell time traces, DBP is found to produce similar nested dynamics to that in the neural tube.

**Figure 9.**
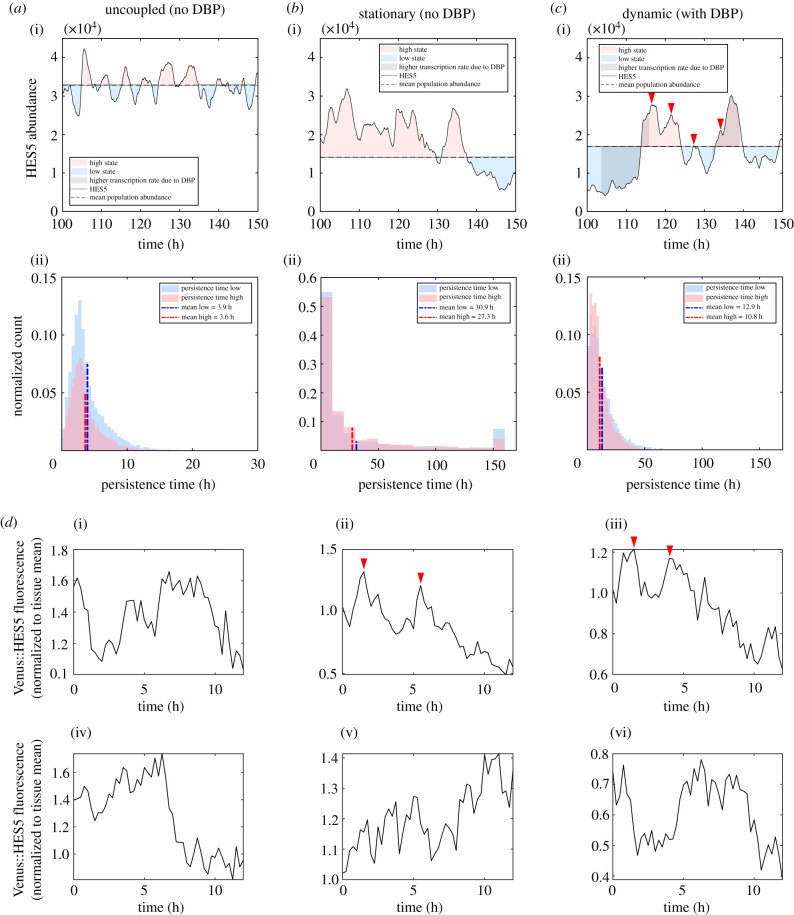
(*a*–*c*), (i) Representative single-cell time traces, (ii) histograms of individual persistence times *T*_↑,*n*_ (pink), and *T*_↓,*n*_ (blue) (see §2.4 for a definition of persistence times). (*a*) Model output with no DBP algorithm included and no LI coupling between the cells. (*b*) The model output with no DBP algorithm included and *P*_0,*LI*_ = 4000. (*c*) The model output with DBP algorithm included and *P*_0,*LI*_ = 4000, and red arrows indicate peaks in ultradian oscillations. Simulations in (i) used 1 column, 26 rows, and simulation run time of 200 h. (ii) Data from 30 individual simulations plotted. Each simulation used 26 rows, 1 column, and simulation run time of 300 h, and only the last 150 h of the simulation was used to measure persistence time. (*d*) (i–vi) Single-cell Venus::HES5 fluorescence time traces taken from experiment source data in [3] and replotted here.

In addition to *D*_*c*_ values, persistence time gives useful information about which mechanisms are contributing to any switching behaviour that is occurring. As defined in §2.4, persistence time refers to the amount of time a cell spends in a high or low state before switching to the opposite fate, and the distribution of low and high persistence times is plotted in figure 9*a*–*c* (ii). Noisy/ultradian dynamics are characterized by mean persistence times of around 3.6–3.9 h, as revealed by running the model without LI and without DBP (figure 9*a* (ii)). These noisy/ultradian persistence times that make up the left-most parts of the distributions are found to be present in all model conditions (figure 9*a*–*c* (ii)).

When LI coupling is included in the simulations, but DBP is not (figure 9*b* (ii)), the distribution is shifted to longer persistence times than in the uncoupled case. The LI induces two larger amplitude high and low states (figure 9*b* (i)), while maintaining smaller amplitude noisy/ultradian oscillations at the mean levels dictated by the LI. This results in noisy/ultradian dynamics not being able to contribute to the switching as much, and the mean persistence times are longer at 27.3–30.9 h. In addition, a peak at 150 h is found (figure 9*b* (ii)), which means that a fraction of the cells spend the entire measurement time stuck in one state, indicating more stationary patterning.

Crucially, the inclusion of DBP into the LI model (figure 9*c*) removes the peak at 150 h, while still maintaining longer mean persistence times (10.8–12.9 h) than the uncoupled model. The single-cell time trace in figure 9*c* (i) shows a more regular switching than in figure 9*b* (i), while still being distinct from the shorter timescale ultradian oscillations in figure 9*a* (i). Relating this back to the observation of nested dynamics in [14], it can be seen that a similar nested oscillation behaviour occurs in the model with DBP included (figure 9*c* (i)), with a mixture of smaller amplitude noisy dynamics and ultradian oscillations (red arrows in 9*c* (i)) being nested within the larger amplitude switching dynamics generated by the LI and DBP algorithm.

While there is more regularity in the switching in figure 9*c* than in figure figure 9*a* or *b*, the distribution still has a wide spread of possible persistence times in figure 9*c* (ii). The *ex vivo* observations are limited to measurement times of at most 16 h, and the mean experimental persistence times indicated a tighter distribution with a mean value of around 8h. While inclusion of DBP in the model fits the data closest, further exploration is needed to understand how the persistence time distributions can be made tighter in line with the *ex vivo* observations.

For a visual comparison between the model with DBP (figure 9*c* (i)) and the experimental data, single-cell time traces are plotted in figure 9*d* (i–vi), which show Venus::HES5 fluorescent intensities tracked over 12 h (replotted from source data in [3]). These traces are limited to 12 h due to experimental constraints, much shorter than the plotted simulation outputs, and so at most show two switches between high and low states (figure 9*d* (i) and (vi)). Both high-to-low and low-to-high switches are observed in the experimental data, with a mix of aperiodic and period dynamics observed. Figure 9*d* (ii) and (iii) show more pronounced transient ultradian oscillations which then become aperiodic noisy expression at later time points (red arrows indicate peaks of ultradian dynamics). Importantly, the amplitude (peak to trough difference in fluorescence) is smaller in the ultradian oscillations than in the long-term switching behaviour of the single cells, and previous analysis found that the long-term trend has an amplitude (standard deviation of normalized HES5 expression levels) approximately twice that of the ultradian oscillations [3]. Though not quantified here, it can be seen in figure 9*c* (i) that the ratio of longer switching to ultradian oscillation amplitude is of the same order as the experimental data.

### 3.5. Dynamic patterning spreads out differentiation events spatially while maintaining a higher differentiation rate than the uncoupled model

**Figure 10.**
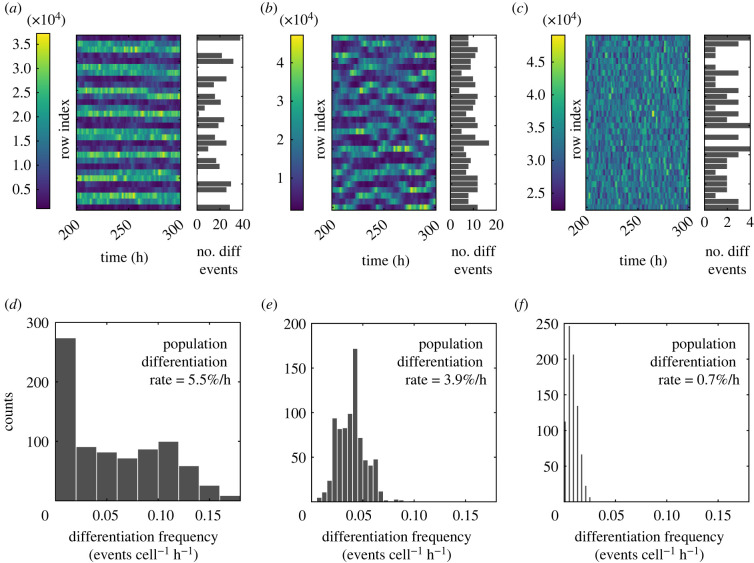
Spatial and temporal measurements of differentiation in three different simulations conditions: (*a*) and (*d*) output from a stationary spatial pattern (no DBP algorithm used), (*b*) and (*e*) a dynamic spatial pattern (DBP algorithm included) and (*c*) and (*f*) show no spatial pattern (uncoupled cells/no lateral inhibition). (*a–c*) The last 100 h of simulation as kymographs and plotted to the right of each kymograph is a histogram showing the total number of differentiation events that occur in each row over the entire 300 h, though only the last 100 h is plotted in the kymographs. (*d–f*) Histograms of the differentiation frequency (§2.5) in individual cells (events cell^−1^ h^−1^), and also the rate of differentiation of the population is given as the a percentage of the population size per hour (800 h of simulation to produce graphs (*d–f*)). All simulation conditions use *P*_*ND*0_ = 3500, *ɛ*_*d*_/*ɛ*_*p*_ = 1.5, with a grid size of 26 columns by 1 row.

To explore the potential functionality of the dynamic patterning, the spatial distribution of differentiation events and the rate of differentiation was explored by comparing three different conditions: a stationary spatial pattern (figure 10*a*,*d*), a dynamic spatial pattern (figure 10*b*,*e*), and no spatial pattern via uncoupled cells (figure 10*c*,*f*).

To visualize how patterning affects the spatial aspect of differentiation, bar graphs showing the total number of differentiation events that occurred over a simulation were plotted to the right of the kymographs in figure 10*a*–*c*. In the stationary patterning case figure 10*a*, it can be seen that the likelihood of differentiating is spatially inhomogeneous. Because low-expressing areas are more likely to incur a differentiation event and because these low-expression regions are fixed in time, the differentiation distribution reflects the periodicity of the pattern. Conversely, the dynamic spatial pattern and no spatial pattern case (figure 10*b* and *c*) have more homogeneous spatial distributions of differentiation events, since every cell can switch between higher and lower expression.

To explore how differentiation rate is affected, the distribution of differentiation frequency in individual cells was plotted (figure 10*d*–*f*), along with the differentiation rate of the population as a percentage. The stationary pattern had the highest rate of differentiation at $5.5\text{\%}\;h^{-1}$ (D), and the distribution of differentiation frequency in individual cells showed a bimodal distribution, which reflects the two different rates of differentiation occurring in the low and high expressing cells. The dynamic pattern showed a slightly lower rate of differentiation compared with the stationary pattern at $3.9\text{\%}\;h^{-1}$, and the distribution has a single peak. The uncoupled no pattern case showed a very low rate of differentiation rate at 0.7% h^−1^, and also with a single peak. See electronic supplementary material, movies S1 and S2 for animated one-dimensional simulations without and with DBP included.

The spatial and temporal measures taken together indicate that for a dynamic pattern, differentiation events are spread out spatially rather than concentrated in one position as is the case in stationary patterns. This spatial spreading of differentiation is also naturally achieved in the case when there is no coupling/no LI; however, in the absence of Notch amplifying the differences between cells, cells do not have such a high amplitude between high and low expression, resulting in a much lower rate of differentiation. Therefore, dynamic patterning maintains a high differentiation rate like that in stationary patterning, but enables a more homogeneous distribution of differentiating cells in space.

## 4. Discussion

In this paper, we have investigated the mechanism and function of dynamic spatial patterns in development, motivated by observations of periodic clusters of HES5 expression that change peak and trough location over time [14]. We introduced two methods that can be used to identify dynamic spatial patterns. First, spatial signals at individual time points can be tested for periodicity using a Fisher *g*-test on the generated power spectra. Second, we proposed the dynamicity coefficient as a measure to test whether peaks and troughs in a spatial signal switch states over time by comparing proportions of time spent in high and low states. Previous models accounted for the observed synchronization of ultradian oscillations between neighbouring cells, but did not capture the generation of three- to four-cell periodicity or the dynamic switching of the spatial pattern.

To address the generation of a three- to four-cell spatial period, we extended the signalling distance in the model by introducing distal cells (figure 6*d*), which generated three- to four-cell periodicity, compared with the two-cell periodicity that occurs in nearest-neighbour signalling. We propose that dynamically extending and retracting protrusions carrying Notch ligands probably account for extended signalling in the neural tube tissue [17,25–28]. If protrusions are the underlying mechanism, then distal signalling efficiency is interpreted as the amount of Notch signalling occurring at the cell body versus at the protrusions. The model predicts that the type of dynamic pattern observed in the neural tube is more likely to occur at higher distal strengths (*ɛ*_*d*_/*ɛ*_*p*_ > 1). As discussed in §3.3, this could be due to differences in mechanical activation, diffusion rates or *cis*-inhibition between the cell body and the protrusions [18,48]. Future experimental work should focus around characterizing the extent and dynamics of protrusions in the developing mouse neural tube, and where Notch and Delta are localized on cell membranes to get a better picture of where Notch signalling is most active.

To understand how dynamic switching of the spatial pattern arises in the neural tube, we explored the potential role of differentiation and dynamic signalling strength. To translate the differentiation process into the model, we implemented a perturbation process where cells that contact a differentiating cell experience an upregulation in HES5 transcription rate (DBP algorithm, outlined in §2.5). The inclusion of DBP in the model resulted in a region of parameter space being identified where dynamic spatial patterning occurs, indicating that with sufficient and regular perturbation, high and low states generated by the underlying Notch LI circuit can be dynamically switched and reorganized. In addition, nested dynamics of ultradian oscillations on top of the larger amplitude switching dynamics were observed in the model-generated single-cell time traces (figure 9), similar to that observed *ex vivo* [14]. One aspect that remains unclear due to experimental limits of the observation time of the *ex vivo* slices, is the regularity of switching. Future experimental work therefore would be very informative if longer observation times could be obtained, as this would enable more detailed comparison of the model-generated persistence time distributions against the data.

Due to higher numbers of signalling interactions in the two-dimensional simulations, regions of dynamic patterning in parameter space were found to be more restricted than in the one-dimensional simulations. From biological studies, the average number of signalling neighbours per RG cell is not known, so whether the one- or two-dimensional simulations are more representative of the biological system is unclear. Some studies suggest that most of the Notch signalling occurs at the apical side of the neuroepithelium [29,49], in which case the restricted number of spatial interactions might be more akin to the one-dimensional model. Other studies show that Delta-carrying protrusions extend down from the basally located newborn neurons to interact with apically located RG cells, and RG cells extend dynamic protrusions in both apical and non-apical locations, in which case the two-dimensional simulations may be more representative of the number of signalling interactions [25,26,28]. An additional consideration is where and when differentiating cells have an altered signalling effect on their neighbours. It would be interesting to introduce cell movement into future modelling so as to investigate the effect of the apical–basal migration of differentiating cells.

It is important to consider that perturbations could reasonably come from sources other than altered signalling in differentiating cells. Processes such as the extension and retraction of signalling protrusions, cell cycle variations in HES5/coupling strength, interkinetic nuclear migration and pulsatile Dll1 signalling are all reasonable candidates in contributing to the switching behaviour [25,28,50–52]. The DBP algorithm is general enough that it could reasonably be adapted to any of the listed alternatives, by altering the magnitude and duration of the perturbation, as well as the parameter it is applied to. Furthermore, there are also entirely separate mechanisms that could underlie the observed HES5 pattern that are not perturbation based. For example, we also explored morphogen gradient-induced travelling waves as a potential mechanism (not included in this study), inspired by somitogenesis studies. We found the travelling waves did not as closely match the data and required assumptions that seemed less likely from the literature, but we cannot rule this mechanism out without further exploration. For the mechanism underlying the clustered/extended spatial periodicity, it may be also worth considering modifications of Notch signalling such as *cis*-inhibition or lateral induction from other Notch ligands such as Jagged, as both of these mechanisms show a tendency to form longer range or clustered patterning [53,54].

Regardless of underlying mechanisms, this is a model that produces a dynamic pattern sufficiently similar to that of HES5 in the neural tube and so we tested what functional advantage such a dynamic pattern might provide during development, finding that dynamic patterning spreads out differentiation events spatially, rather than generating hotspots of differentiation like in the stationary case. Stationary patterning seems most suited to tissues where differentiating cells remain within the progenitor population and need to be regularly spaced apart such as in the formation of sensory hairs in *Drosophila* [19]. In the developing neural tube, differentiating cells do not form a regular pattern of differentiated cells within the progenitor population itself, rather they leave the progenitor population and migrate basally to form neurons and glia at later stages [55]. Although the functional advantage of dynamic patterning is not established, we conjecture that it ensures that the production of neurons is evenly distributed across the dorsal–ventral axis and prevents many differentiating cells from being repeatedly produced in the same locations as in the stationary patterning case.

In sum, we have explored how a stationary pattern generating signalling network, Notch LI, can be made dynamic through the introduction of perturbations that enable cells to switch between high and low expression. We suggest that a combination of protrusions and altered signalling strength coming from differentiating cells are the most likely underlying mechanisms that produce the dynamic HES5 spatial pattern found in the developing neural tube. However, further experiments need to be carried out regarding the presence of Notch carrying protrusions, and whether these protrusions are capable of generating extended spatial periodicity, along with tests of how much perturbation comes from differentiating cells in the developing neural tube.

## Data Availability

All code is written in Matlab and is available at https://github.com/Papalopulu-Lab/Hawley2022. The data are provided in electronic supplementary material [56].
